# Evaluation Methods for Water Resource Suitability in Territorial Spatial Planning: A Case Study of Baiyin City in a Semi-Arid Region

**DOI:** 10.3390/ijerph191912973

**Published:** 2022-10-10

**Authors:** Jiuyi Li

**Affiliations:** Institute of Geographic Sciences and Natural Resources Research, Chinese Academy of Sciences, Beijing 100101, China; lijiuyi@igsnrr.ac.cn

**Keywords:** territorial spatial planning, suitability of water resources for agricultural production (WRSA), suitability of water resources for urban construction (WRSU), “Double Evaluation”, Geodetector, arid and semi-arid area, Baiyin City

## Abstract

Water resources are a major factor in the spatial layout of agricultural production and urban construction, which is an important part of China’s ongoing territorial spatial planning. In order to assess the constraining and guiding effects of water resources on territorial spatial planning, water resources suitability evaluation needs to be carried out at the grid scale. Traditional basin or regional-scale indicators of water resources cannot satisfy the requirements with high spatial accuracy in territorial spatial planning, because the internal differences could not be described. In this study, irrigation water supply cost index (CIA) and urban water supply cost index (CIU) were evaluated to characterize the affordability of potential water supply costs by simulating of optimal water supply path. Further, grid-scale indexes of water resource suitability for agricultural production (WRSA) and for urban construction (WRSU) were constructed. The grades of WRSA and WRSU were classified at a 20 m grid scale in Baiyin City. The areas of water resources that were suitable, relatively suitable, less suitable, and unsuitable for agricultural production were 381.0 km^2^, 3354.7 km^2^, 3663.9 km^2^, and 12,700.7 km^2^, respectively, accounting for 1.9%, 16.7%, 18.2%, and 63.2% of the total area of Baiyin City. The areas of water resources that were suitable, relatively suitable, less suitable, and unsuitable for urban construction were 1657.7 km^2^, 4184.5 km^2^, 1177.7 km^2^, and 13,075.7 km^2^, respectively, accounting for 8.2%, 20.8%, 5.9%, and 65.1% of the total area of Baiyin City. Coupling analysis with land use and land resources suitability were carried out in this study, which showed that the grid-scale WRSA and WRSU could well characterize the spatial differences of water resources suitability for agricultural production and urban construction. The results of the Geodetector-based study show that the WRSA and WRSU indicators have better explanatory power for the land-use spatial distribution compared to indicators such as water distance. Therefore, the indexes could provide scientific support to delimit agricultural space and urban space, and are effective means of “determining regional functions by water resources“ in territorial spatial planning. Furthermore, the indexes could be applied to other arid and semi-arid areas, and also hilly areas, where water supply suitability plays a restrictive role in agricultural production and urban construction.

## 1. Introduction

Water resources are a limiting factor for agricultural production and urban construction in arid and semi-arid regions [1,2]. To describe the ability of water resources to support regional development, many indicators and methods have been proposed. Per capita water resources are used to assess water stress on a national scale [3,4]. However, this indicator does not effectively describe regional-scale water resources conflicts due to the great differences in industrial structure and water use efficiency among regions. The water resources withdrawal rate can characterize the water resource potential for regional development, and many indicators based on this are constructed to assess the degree of regional water scarcity and its vulnerability [5,6,7,8,9,10,11,12,13]. China is a very water-scarce country, and with rapid economic development, the conflict between water supply and demand is becoming more and more prominent. The ability of water resources to support populations and economic development is assessed and used to guide regional development decisions in water-scarce areas [14,15,16,17,18,19,20]. In 2010, China released the Major Function Oriented Zoning, which divides the major functions of ecological protection, urban development, and agricultural production for 2844 county units. Li et al. evaluated the ability of water resources to support population concentration and industrial development, and the results on the county scale provide a scientific basis for the formulation of the Major Function Oriented Zoning [21].

In 2019, China began to compile territorial spatial planning for 31 provinces, 333 cities, and 2844 counties, integrating three types of spatial plans: Major Function Oriented Zoning, land use planning, and urban and rural development planning. The territorial spatial planning delineates the spatial scope of ecological space, agricultural space, and urban space, and delineates the ecological protection red line, permanent basic agricultural land, and urban development boundary, playing a fundamental role in China’s planning system. The government proposes to take the evaluation of resources and environmental carrying capacity and the evaluation of territorial space development suitability (referred to as “Double Evaluation”) as the basis for the preparation of territorial spatial planning. In “Double Evaluation”, resource and environmental conditions for agricultural production and urban construction need to be assessed, such as water resources, land resources, environment, ecology, geological hazards, etc. In order to guide the scientific evaluation of the natural conditions, the Guidelines for the Evaluation of Resources and Environment Carrying Capacity and the Evaluation of Territorial Space Development Suitability (referred to as “Guidelines”) was issued by the Ministry of Natural Resources in January 2020. According to the “Guidelines”, water resources need to play the role of “determining city scale, regional function, population amount, and industrial types” in territorial spatial planning. Water resources conditions vary greatly from region to region in China, so the “Guidelines” only gives the framework of the evaluation methods. Therefore, water resources evaluation indicators, parameters, and grading thresholds need to be studied in practice to suit the needs of planning [22]. Two studies need to be completed in water resources evaluation. On the one hand, the water resources carrying capacity should be evaluated, which constrains the total amount of irrigated agriculture and urban population. On the other hand, the spatial difference of water resources suitability should be evaluated, which provides guidance for the spatial layout of agricultural production and urban construction.

The theory and methods of water resources carrying capacity research are relatively well developed. In the early 1980s, UNESCO put forward the concept of “resource carrying capacity”, which was defined as the number of people that a country or region can sustainably support at a certain material standard of living within a foreseeable period by using local natural resources and technological conditions [23]. Water resources carrying capacity is an important component of natural resources carrying capacity, which refers to the maximum supporting capacity of regional water resources for social and economic development within a certain stage of social history and scientific and technological development, under the condition of maintaining ecology and environmental health. Chinese experts have carried out many empirical studies in arid and semi-arid areas, where water demand for agricultural and urban development is predicted and water availability is assessed. On this basis, the reasonable capacity of water resources that can carry population and economic development guides the layout of regional agricultural production and town construction [24,25,26,27,28].

The study of water resource suitability for regional development was first applied to agro-climatic zoning studies [29,30,31]. Scholars have discussed the impact of different water resource conditions on agricultural production and urbanization, mainly assessing regional-to-regional differences [32,33,34,35,36,37,38,39]. However, regional indicators cannot describe the differences within a region and cannot meet the high precision requirements of terrestrial spatial planning [40,41,42,43]. In the spatial variability analysis of water resources suitability, water source distance is usually used to characterize the accessibility factor [44,45,46,47,48,49]. However, the distance to the water supply source does not equate to water accessibility, especially in areas with complex topographical conditions [50,51]. Water resource condition is the restrictive factor for the spatial layout of agricultural production and urban construction, especially in arid and semi-arid areas [52,53,54,55]. In territorial space planning, it is difficult for “determining regional function by water resources” without internal variation assessment of water resources suitability. Constructing a grid-scale water resources suitability index and scientifically evaluating the spatial difference of water resources suitability is a practical problem in the “Double Evaluation” of territorial spatial planning [56,57,58].

Therefore, Baiyin City, which is located in a semi-arid area with complex topographic conditions, was selected as a case area. In this study, grid-scale water resources suitability indicators and models were constructed, and the suitability levels of water resources suitability for agricultural production and urban construction in Baiyin City were classified. The results can provide a scientific basis for the division of urban space and agricultural space in the territorial spatial planning of Baiyin City, and also provide a reference for “Double Evaluation” in territorial spatial planning in other regions.

## 2. Materials and Methods

### 2.1. Study Area

**Figure 1:**
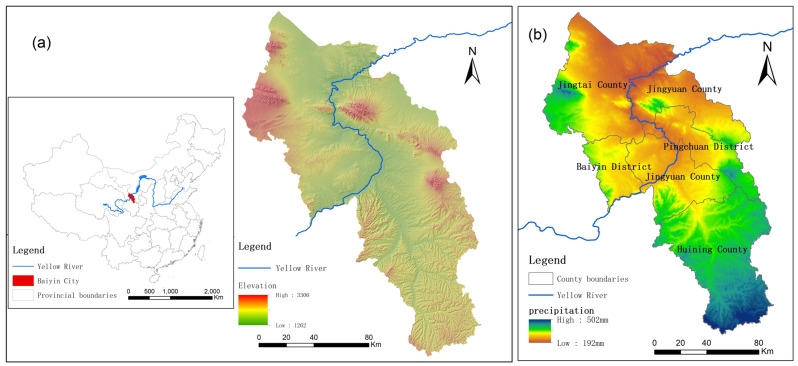
(**a**) Location of Baiyin City; (**b**) Average annual precipitation.

Baiyin City, Gansu Province, is in the upper reaches of the Yellow River Basin (Figure 1a), in the transition zone between the Loess Plateau and Tengger Desert. Baiyin has a continental monsoon climate, with precipitation decreasing from south to north. The average annual precipitation in Baiyin City was 319 mm (Figure 1b). The spatial variation of precipitation is significant, reaching 500 mm in the south and less than 200 mm in the north. Because most areas of Baiyin City are in semi-arid areas, water scarcity has become a limiting factor for regional development. Baiyin City is divided into two districts and three counties, namely Baiyin District, Pingchuan District, Huining County, Jingyuan County, and Jingtai County.

**Figure 2:**
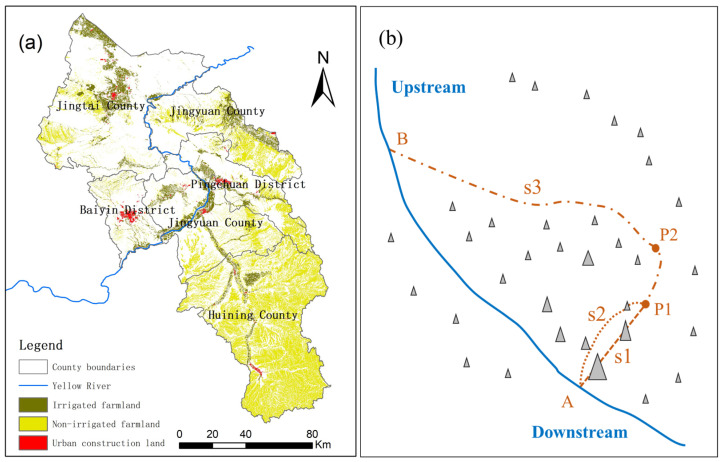
(**a**) Distribution of cultivated land and urban construction land; (**b**) difference between water source distance and water accessibility.

The Third National Land Survey showed that the cultivated land area of Baiyin was 5751.2 km^2^ in 2018, accounting for 27.1% of the total land area (Figure 2a). Among them, the area of non-irrigated farmland was 4456.3 km^2^, accounting for 77.5% of the total cultivated land area. The irrigated farmland area was 1294.9 km^2^, accounting for 22.5% of the total cultivated land area. Baiyin has a total population of 1.74 million, including 0.88 million in the urban population, with an urbanization rate of 50.6%. The urban construction land area is 141.7 km^2^, mainly concentrated in the central city and several towns. The spatial distribution of the cultivated land and urban construction land is shown in Figure 2a. Water resources per capita in Baiyin City are only 113 m^3^, which is extremely scarce. In 2018, the total water consumption of Baiyin City was 905 million m^3^, 4.6 times the average annual local water resources. Due to a serious shortage of local water resources, agricultural irrigation and urban development mainly rely on the Yellow River and on water transfer projects, such as the Xingdian, Jingdian, Jinghui, and Wuchuan trunk canals. According to the “Three Red Lines” of Gansu Province, the total water consumption control target of Baiyin City in 2020 and 2030 was 1.079 billion m^3^ and 1.243 billion m^3^, respectively, which was 1.19 times and 1.37 times the water consumption in 2018. Therefore, there is still a certain potential for development and utilization.

According to the “Guidelines”, indexes of water resources include the utilization ratio of water resources, the average per capita water resources, and precipitation, etc. The utilization ratio of water resources and average per capita water resources data are available only in county units. All the counties in Baiyin City are extremely water-scarce, and the spatial variability of water resources conditions cannot be characterized. Because agricultural irrigation and urban water supply in Baiyin City mainly rely on water diversion projects, water supply cost is a key factor for the water resources suitability on a spatial scale. There are many hills and terraces in Baiyin City that need to pump water from the supply sources. Therefore, the distance from the water supply sources cannot directly describe the cost of the water supply, which is typical in the arid and semi-arid areas of Northwest China. Using Figure 2b as an example, the optimal water supply path from water source A to P1 may be the curve s2 rather than the straight line s1, because curve s1 has fewer hills in its path. Furthermore, the water supply path s3 from farther B may be preferable to path s2. Moreover, water resources suitability at point P2 is better than that at point P1, even though P2 is further away from the river source in a straight line. In order to solve this problem, we used the cost distance method in Arcgis to simulate the optimal water supply path with the lowest water supply cost for each raster. Based on the assessment of potential water supply costs, a water resource suitability index was constructed to characterize the spatial variability in the suitability for agricultural production and urban construction. In particular, it should be noted that the suitability for agricultural production and urban construction are different. Firstly, some agricultural irrigation water supply sources cannot meet the needs of urban development. Secondly, urban development can afford higher water supply costs, i.e., longer water supply distances and greater lifting heights from the water source. Therefore, the water source selection and parameters for water supply cost are different in the suitability evaluation of the two functions.

### 2.2. Data Sources

The 1:50,000 scale land use vector map came from the Third National Land Survey in 2020. DEM data in a 30 m grid were downloaded from the Resource and Environment Data Center, Chinese Academy of Sciences (https://www.resdc.cn (accessed on 1 October 2022)). All the above data were resampled to a 20 m grid using the bilinear interpolation algorithm in ArcGIS. The precipitation and evaporation data of meteorological stations in and around Baiyin City from 1956 to 2018 were derived from the National Meteorological Information Centre (https://data.cma.cn (accessed on 1 October 2022)) and were interpolated to a 20 m grid using the Kriging method. Vector data of rivers and canals were provided by the Baiyin Natural Resources Bureau.

### 2.3. Runoff Model Construction and Water Supply Sources Extraction

Agricultural and urban water supply sources include rivers, reservoirs, and canals. There are only a few small- and medium-sized reservoirs, which were built on the rivers. Therefore, only river sources and canal sources were analyzed in the evaluation of water supply cost, and the reservoirs were considered as a part of the river sources. The field investigation of water supply in Baiyin City showed that the river’s annual average runoff of more than 5 million m^3^ can meet the water requirements of agricultural water supply sources in irrigated areas. Rivers with an annual average runoff of more than 25 million m^3^ can meet the water requirements of small- and medium-sized towns. A 20 m grid-scale runoff and concentration model of Baiyin City was constructed to extract the potential river sources that meet the water supply requirements.

In the runoff and concentration model, runoff was obtained by subtracting actual evaporation from precipitation, which was calculated using Zhang Lu’s formula [59,60]:

(1) $$\frac{E}{P}=\frac{1+\omega \frac{E_{0}}{P}}{1+\omega \frac{E_{0}}{P}+{\left(\frac{E_{0}}{P}\right)}^{-1}}$$

where *E* is the actual evapotranspiration, *P* is precipitation, *E*_0_ is potential evapotranspiration, and *ω* is the plant-available water coefficient.

**Figure 3:**
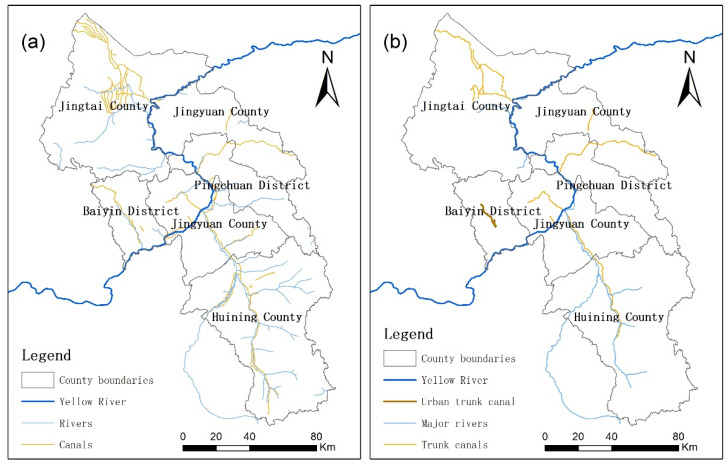
(**a**) Irrigation water supply sources; (**b**) Urban water supply sources.

After the steps of DEM filling, slope aspect analysis, grid runoff analysis, flow accumulation, and river extraction meeting the requirements of flow conditions, potential river sources for agricultural and urban water supply in Baiyin City were distinguished. Combined with the canal sources, the potential water supply sources for irrigation and for urban construction in Baiyin City were shown in Figure 3a,b. The extracted river sources matched well with the river data provided by Baiyin Natural Resources Bureau. A runoff factor was considered in the model, so the extracted river sources were more well-fit to the actual water sources, which was confirmed in the field investigation. Among the water supply sources for agricultural irrigation, the water supply assurance rate of the Yellow River is relatively high, and the other river and canal sources are relatively low. Among the water supply sources for urban construction, the water supply assurance rate of the Yellow River and central city trunk canal are relatively high, and the other river and canal sources are relatively low.

### 2.4. Water Resource Suitability Indexes Construction

#### 2.4.1. Suitability of Water Resources for Agricultural Production (WRSA)

Water scarcity is an important constraint to agricultural production, mainly in two aspects: the supporting capacity of precipitation, and the supporting capacity of water supply for irrigation. The supporting capacity of water supply for irrigation refers to the reliability of irrigation water sources and the cost of irrigation water supply. The reliability of irrigation water sources is mainly related to the amount of water and the guarantee rate of irrigation water supply. In other words, the larger the amount of water and the higher the guarantee rate, the stronger the reliability. The cost of irrigation water supply is mainly related to the distance and lifting height of the water source. The closer distance and smaller lifting height make the cost of irrigation water supply lower.

The irrigation water supply cost index (*CIA*) was measured as follows:

(2) $$CIA=\frac{\sum_{i}d_{Ai}}{D_{Amax}}+\frac{\sum_{i}h_{Ai}}{H_{Amax}}$$

where *CIA* is the water supply cost index for irrigation; *i* refer to the point on the optimal water supply path, just like curve s3 instead of curve s1 in Figure 2b, which is simulated by the path distance module in ArcGIS; $d_{Ai}$, $h_{Ai}$ refer to the distance and the lifting height of the pathway point *i*; and $D_{Amax}$, $H_{Amax}$ refer to the maximum water transfer distance and lifting height that the irrigated agriculture can bear, which were taken as 20 km and 15 m in the evaluation, according to the field survey of the irrigation area in Baiyin City. The criteria shown in Table 1 were used to classify the suitability grades of water resources for agricultural production (WRSA) in Baiyin City. The irrigation water sources were divided into two types according to reliability: the mainstream of the Yellow River and other water supply sources. The CIAs of different sources were calculated using formula 2. In the areas with the Yellow River as a water supply source, WRSA were classified into two types (i.e., suitable and relatively suitable). In the areas with other rivers and canals as irrigated water supply sources, WRSA was also divided into two types (i.e., relatively suitable and less suitable). CIA refers to the ratio of potential water supply costs to affordable costs for agriculture customers, and the value range is [0, 1]. The higher the value of CIA, the worse the accessibility of water supply. When the value is 0, it means that the water source is close and the cost of water extraction is 0. When the value is 1, it means that the potential water supply cost has reached the maximum acceptable level. When the CIA is greater than 1, it means that the potential water supply cost is unaffordable, i.e., irrigation is not available, so the area will be reclassified as being either a less suitable or unsuitable region, based on the average annual precipitation.

#### 2.4.2. Suitability of Water Resources for Urban Construction (WRSU)

Water resources is an important factor influencing urban construction in Baiyin City, mainly reflected in the supporting capacity of water supply for urban development. The supporting capacity refers to the reliability of urban water supply sources and urban water supply cost. The larger the water quantity and the higher the guarantee rate, the stronger the reliability. Additionally, the cost of urban water supply is lower if there is a closer distance and a smaller height of water lifting. This study constructed the water resources suitability index for urban construction, to assess the support and guarantee ability of water resources conditions for urban construction in Baiyin City.

The urban water supply cost index (*CIU*) was measured as follows:

(3) $$CIU=\frac{\sum_{j}d_{Uj}}{D_{Umax}}+\frac{\sum_{j}h_{Uj}}{H_{Umax}}$$

where *CIU* is the water supply cost index for urban construction; *j* refers to the point on the optimal water supply path, simulated by the path distance module in ArcGIS; $D_{Uj},\;H_{Uj}$ refer to the distance and the lifting height of the pathway point *j*, respectively; and $D_{Umax}$, $H_{Umax}$ indicate the maximum water transfer distance and lifting height that the urban development can bear, which were taken as 40 km and 40 m in the evaluation, according to the field investigation of urban water supply projects in Baiyin City.

The criteria shown in Table 2 were used to classify the suitability grades of water resources for urban construction (WRSU) in Baiyin City. The urban water supply sources in Baiyin City were divided into two types: the mainstream of the Yellow River and the central city water supply trunk canal, and other urban river and canal sources. The CIUs of different sources were calculated using Formula (3). In the areas with the Yellow River and the central city water supply trunk canal as the water supply source, WRSU were classified into two types (i.e., suitable and relatively suitable). In the areas with other rivers and canals as urban water supply sources, WRSU was also divided into two types (i.e., relatively suitable and less suitable). CIU refers to the ratio of potential water supply costs to affordable costs for urban customers, and the value range is [0, 1], the same as CIA. Areas with a CIU of larger than 1 were classified as being unsuitable.

### 2.5. Geodetector

Geodetector is a method to measure the correlation of spatial distribution of different factors at various scales [61,62], and is widely used in spatially stratified heterogeneity (SSH) studies [63,64,65,66,67,68,69,70], which can effectively identify the spatial differentiation of geographic phenomena and their influencing factors. The software of Geodetector can be downloaded for free at http://www.geodetector.cn/ (accessed on 1 October 2022). The principle of the method is as follows:

(4) $$q=1-\frac{SSW}{SST}=1-\frac{\sum_{h=1}^{L}N_{h}\sigma_{h}^{2}}{N\sigma^{2}}$$

where *SST* is Total Sum of Squares; *SSW* is Within Sum of Squares; the *q*-statistic measures the association between *X* and *Y*, both linearly and nonlinearly. The value of *q* is strictly within [0, 1]. If *Y* is stratified by an explanatory variable *X*, then *q* = 0 indicates that there is no coupling between *Y* and *X*; *q* = 1 indicates that *Y* is completely determined by *X*; *X* explains 100*q*% of *Y*.

The Geodetector method was used to assess the explanatory power of the indicators constructed in this study on the currently cultivated land and construction land. Moreover, the comparison with traditional indicators and the coupling analysis with land resources indicators were carried out.

## 3. Results

### 3.1. WRSA

**Figure 4:**
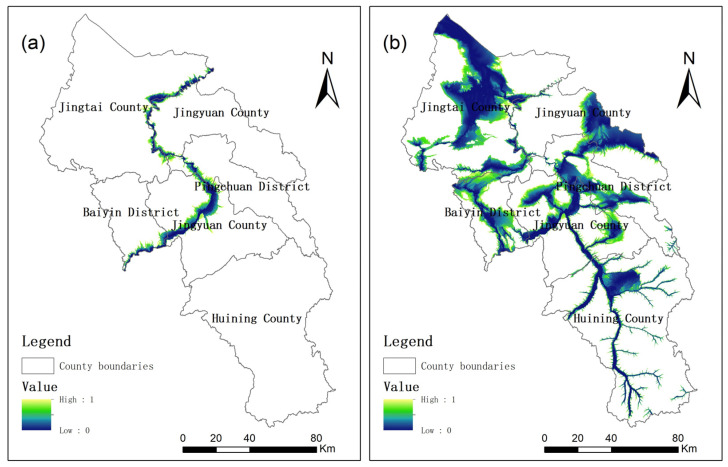
(**a**) CIA of the Yellow River; (**b**) CIA of other agricultural water supply sources.

The CIAs of the Yellow River and other agricultural water supply sources are shown in Figure 4a,b. The index decreases with the distance from the water source. However, the decreasing rate differs significantly, due to different topographic conditions. The suitable range is wider in the areas with flatter terrain. Obviously, CIA better represents the water supply cost than the river distance.

**Figure 5:**
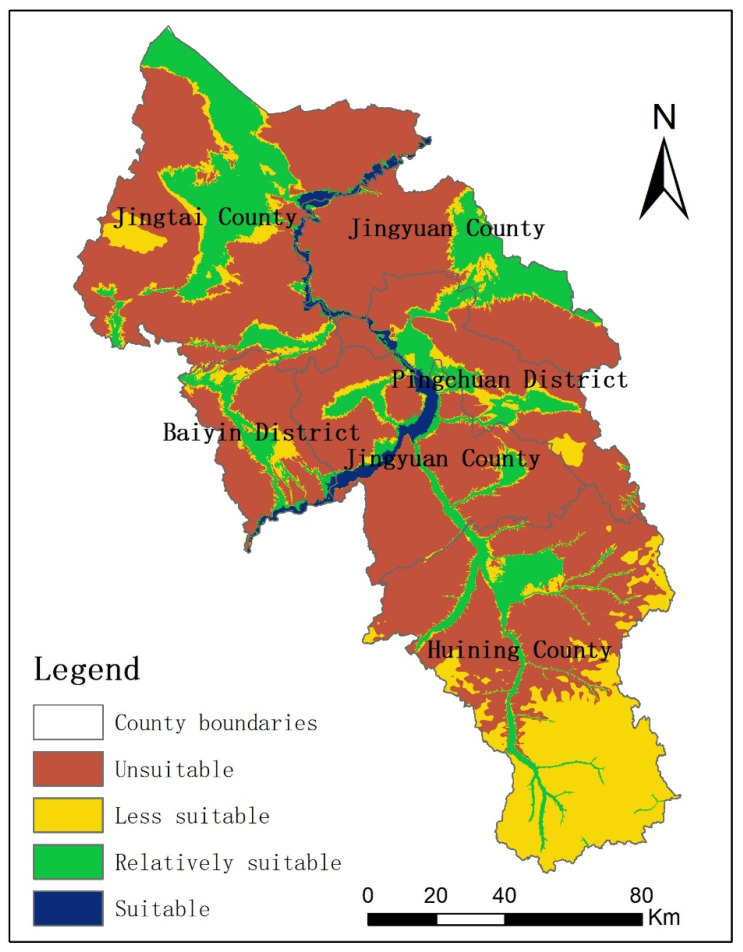
WRSA of Baiyin City.

According to the discriminant matrix shown in Table 1, the WRSA grades of Baiyin City were divided, and the result is shown in Figure 5. The areas that were suitable, relatively suitable, less suitable, and unsuitable for agricultural production were 381.0 km^2^, 3354.7 km^2^, 3663.9 km^2^, and 12,700.7 km^2^, accounting for 1.9%, 16.7%, 18.2%, and 63.2% of the total area of the city, respectively. The suitable areas were concentrated in the Yellow River valley area, with good water supply convenience. The suitable area was small, due to the large slope in the valley area. Relatively suitable areas were mainly concentrated in the Yellow River valley and the Zuli River valley, as well as the Xingdian, Jingdian, Jinghui, and Wuchuan irrigation areas. There are two types of less suitable areas. One is distributed around the river valley and irrigation area. The other is in the south of Huining County, with high precipitation, which can meet the requirements of non-irrigated farmland. Unsuitable areas were mainly located in the central and northern parts of Baiyin City, where both the precipitation and irrigation conditions are poor.

The distribution of cultivated land under different WRSA grades is shown in Table 3. The cultivated land areas accounted for 35.1%, 39.6%, 46.9%, and 20.2% of the suitable area, relatively suitable area, less suitable area, and unsuitable area, respectively, and the proportion of irrigated farmland in the cultivated land were 98.4%, 71.0%, 4.9%, and 5.3%. Irrigated farmland was mainly distributed in the suitable area and relatively suitable area, which indicates that the evaluation results of WRSA well described the spatial differentiation of water resource suitability.

### 3.2. WRSU

**Figure 6:**
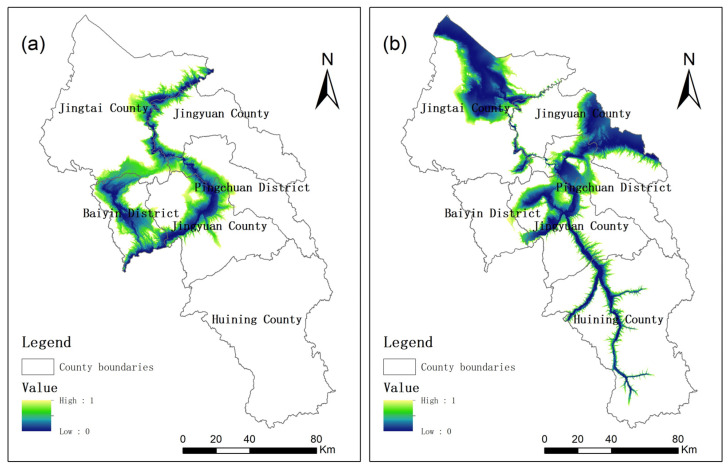
(**a**) CIUs of the Yellow River and the central city trunk canal; (**b**) CIUs of other sources.

The CIUs of the Yellow River and the central city trunk canal water sources are shown in Figure 6a. CIUs of other urban water supply sources are shown in Figure 6b. The urban water supply range was larger than agricultural water supply range from the same supply source, because urban water users can bear higher costs. At the same time, some areas that are suitable for agricultural production were not suitable for urban construction, because urban development requires a higher quality of water supply sources.

**Figure 7:**
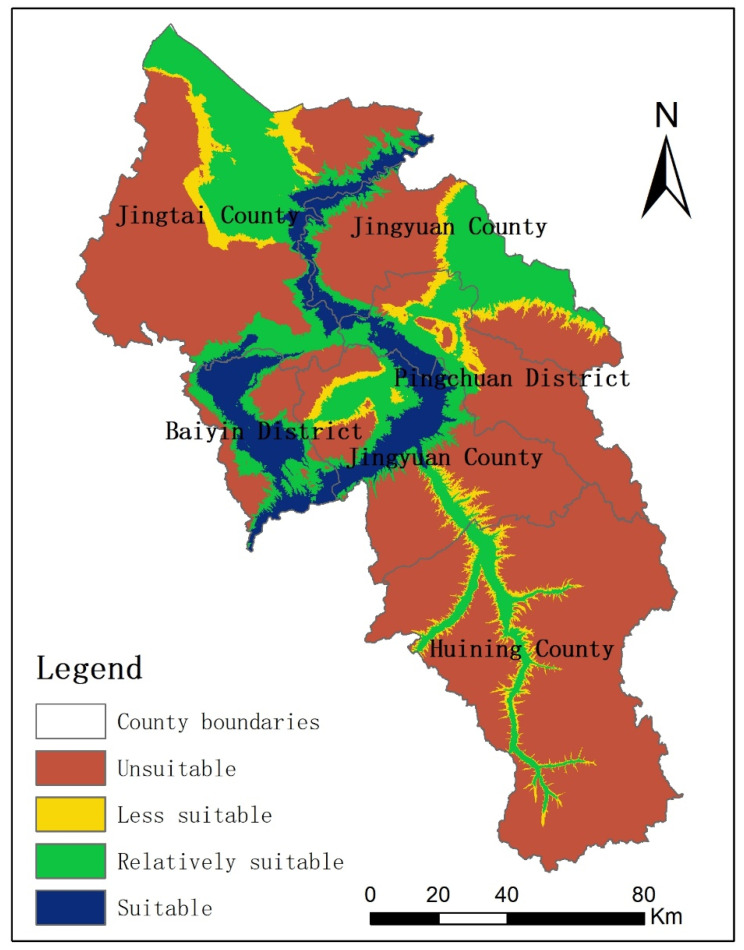
WRSU of Baiyin City.

According to the discriminant matrix shown in Table 2, WRSU grades of Baiyin City were classified, and the results are shown in Figure 7. The areas of suitable, relatively suitable, less suitable, and unsuitable for urban construction were 1657.7 km^2^, 4184.5 km^2^, 1177.7 km^2^, and 13,075.7 km^2^, accounting for 8.2%, 20.8%, 5.9%, and 65.1% of the total area of the city, respectively. The suitable area and relatively suitable area were concentrated in the Yellow River valley and Zuli River valley, as well as the Wuchuan, Xingdian, Jingdian projects water receiving areas. Most rivers in Baiyin City cannot meet the urban water supply requirement, and so the proportion of unsuitable areas was as high as 65.1%.

The distribution of constructed land under different WRSU grades is shown in Table 4. The constructed land areas accounted for 3.3%, 1.6%, 0.3%, and 0.1% of the suitable area, relatively suitable area, less suitable area, and unsuitable area, respectively. A total of 87.5% of the constructed land was concentrated in the suitable area and relatively suitable area. Only 12.5% of constructed land was distributed in the less suitable area and unsuitable area, and mainly consisted of industrial and mining, and storage and commercial land. The high match degree between constructed land and WRSU indicated that the index is a good characterization for urban construction and can be used as the basis for urban space delimitation in territorial spatial planning.

### 3.3. Coupling Analysis with Suitability of Land Resource

According to the “Guidelines”, water resources and land resources are the basic factors for evaluating the suitability of agricultural production and urban construction, playing a decisive role in “Double Evaluation”. The suitability of land resources for agricultural production was classified according to the slope. The areas of 0–2°, 2–6°, 6–15°, 15–25°, and above 25° were divided into suitable area, relatively suitable area, moderately suitable area, less suitable area, and unsuitable area. Irrigated farmland densities (i.e., proportion in the total land area) under different water and land resources suitability grades were calculated, and the results are shown in Table 5. In each land resource suitability grade, the better the WRSA, the higher the irrigated farmland density. For example, the irrigated farmland density was 6.75% in the relatively suitable area of land resources. Among them, the irrigated farmland density within the suitable, relatively suitable, less suitable, and unsuitable grades of WRSA were 24.89%, 19.70%, 4.34%, and 2.29%, respectively. Similarly, in each WRSA grade, the better the land resources suitability, the higher the irrigated farmland density. For example, the average irrigated farmland density in the areas with suitable WRSA grade was 28.14%. Among them, for the irrigated farmland densities within the land resources suitability grades of suitable, relatively suitable, moderately suitable, less suitable, and unsuitable, the cultivated land density was 41.86%, 19.70%, 4.94%, 0.97%, and 0.46%, respectively.

The suitability of land resources for urban construction was classified according to the slope. The areas of 0–3°, 3–8°, 8–15°, and above 15° were divided into suitable area, relatively suitable area, less suitable area, and unsuitable area. Constructed land density (i.e., proportion in the total land area) under different water and land resources suitability grades was calculated, and the results are shown in Table 6. Similarly, construction land density is large in the better WRSU and better land resources suitability.

**Figure 8:**
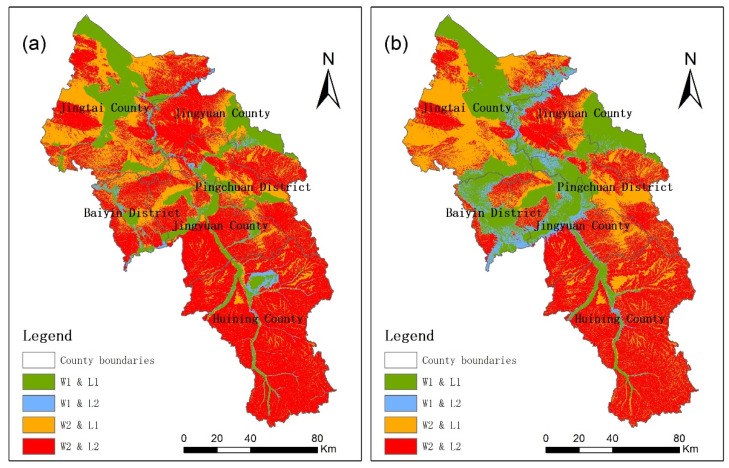
(**a**) Coupling of water and land resources suitable grades for agricultural production; (**b**) Coupling of water and land resources suitable grades for urban construction. W1: water resources suitable area and relatively suitable area; W2: water resources less suitable area and unsuitable area; L1: land resources suitable area and relatively suitable area; L2: land resources moderately suitable area, less suitable area, and unsuitable area.

The suitabilities of water resources and land resources in Baiyin city do not match in space. The overlap results of the two indicators are shown in Figure 8. A total of 62.3% of good suitable grades (i.e., suitable and relatively suitable) of land resource for agricultural production do not have good WRSA grades. On the other hand, 16.2% of good WRSA grades do not have good land resources with suitable grades for agricultural production. The two proportions for urban construction function were 56.4% and 22.0%, respectively. About 60% of the area with good land resources condition were not suitable for agricultural production and urban construction, due to poor water resources suitability. Therefore, WRSA and WRSU can provide more accurate spatial direction in delimiting agricultural space and urban space. Land resources factors are also irreplaceable, because there were many steep slope areas in the water resources suitable area.

### 3.4. Interaction Detection Results by Geodetector

**Figure 9:**
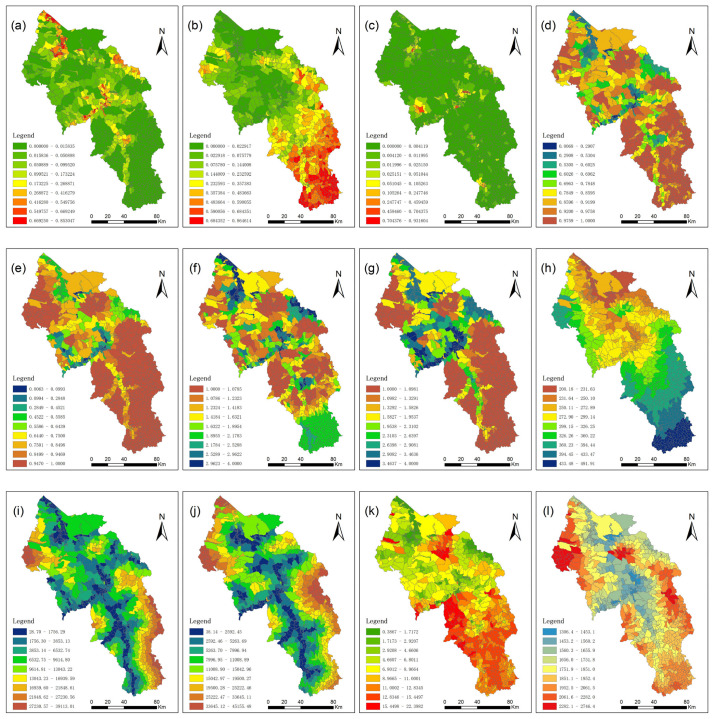
(**a**) Irrigated farmland density; (**b**) non-irrigated farmland density; (**c**) construction land density; (**d**) CIA; (**e**) CIU; (**f**) WRSA, assign values 1–4 for the four suitability grades; (**g**) WRSU, assign values 1–4 for the four suitability grades; (**h**) precipitation; (**i**) distance to irrigation water supply source; (**j**) distance to urban water supply source; (**k**) slope; (**l**) elevation.

**Figure 10:**
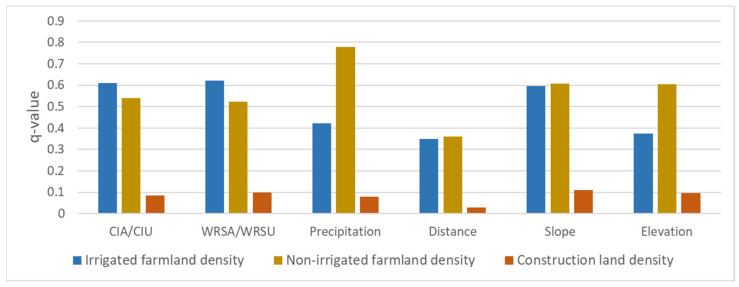
The q-statistic of water and land resources indicators influencing cultivated land and construction land spatial distribution.

The density of cultivated land and construction land, water resources, and land resources indicators were counted for 828 villages in Baiyin City (Figure 9). The q-values for the spatial distribution of irrigated farmland, non-irrigated farmland, and construction land were calculated by Geodetector, and the results are shown in Figure 10. WRSA can explain 62.2% of the spatial distribution of irrigated farmland, which is the highest among all factors. It is followed by CIA and slope with q-values of 0.610 and 0.597, respectively. The results indicate that irrigated farmland in Baiyin City is mainly distributed in areas with convenient water supply and flat terrain, which is consistent with the results of statistical analysis. The q-values of precipitation and elevation are 0.422 and 0.375, respectively, which have weaker effects on the spatial distribution of irrigated farmland than WRSA, CIA, and slope. The distance from irrigated water source can only explain 34.8% of the distribution of irrigated farmland, which is significantly lower than CIA and WRSA, also indicating that the indicators constructed in this study are more effective. Among the factors influencing non-irrigated farmland, precipitation had the highest q-value of 0.779. The q-values of CIA and WRSA for non-irrigated farmland are 0.541 and 0.523, respectively, which are lower than the ability to explain irrigated farmland distribution. It is indicated that non-irrigated farmland in Baiyin City is less affected by water supply conditions and is mainly constrained by precipitation conditions. Slope and elevation also have a strong influence on non-irrigated farmland distribution, with q-value reaching 0.607 and 0.604, respectively. This is mainly because Baiyin City is located in the Loess Plateau area, where agricultural production is strongly constrained by terrain conditions. Among all the six factors, distance from water supply sources has the least explanatory power, the same as that of irrigated farmland. The q-values of the six influencing factors of the spatial distribution of urban construction land are not high. This is mainly due to the small size of urban construction land, which is not subject to strong spatial constraints.

In the detection of factors influencing the spatial distribution of the three land use types, the indicators constructed in this study all showed stronger explanatory power than water distance, indicating that the method is effective and applicable. The spatial constraint of construction land is smaller, so further interaction detection analysis was conducted, and the results are shown in Table 7. The q-value of interaction factors of WRSU and slope is bigger than the sum of q-value of WRSU and q-value of slope, and the same is true for the interaction detection of WRSU and elevation. The results show that the construction land is distributed in areas with superior water resource suitability and superior land resource suitability, and the coupling of the two is better than using them separately. In other words, urban construction activities are usually located in areas where the suitability of both water and land resources is high. In territorial spatial planning, urban spaces should continue to be designated in areas with better water and land resources conditions. In fact, this is the working framework of “Double Evaluation” in territorial spatial planning, and this study verifies the scientific validity of this framework.

## 4. Discussion

### 4.1. Advantages of the Indicators

Because water resources have the characteristics of fluid and dispatchability, the suitability of water resources is more oriented to a region rather than a point. Therefore, most traditional studies on the relationship between water resources and regional development is to evaluate the supporting ability for further concentration of population and industry. Water resources indicators are mostly on a regional scale, such as the amount of water resources per capita and the utilization rate of water resources. In the territorial spatial planning of cities and counties, it is necessary to carry out intra-regional water resources suitability variation assessment, which poses new challenges to water resources research. Therefore, in this study, WRSA and WRSU were constructed, and an empirical study was conducted in Baiyin City as an example. These indicators are jointly measured by the lift and delivery distances under the optimal water supply path simulated by Arcgis, which connotes the ratio between the potential water supply cost and the affordable cost. The results of spatial statistical analysis and Geodetector show that WRSA and WRSU can better characterize water resource suitability differences and have a significant advantage over indicators such as distance to water source. The reason is that in mountainous and hilly areas, where the elevation of farmland and population is much higher than that of water sources, lifting water from valleys requires high costs, and the straight-line distance to water sources does not reflect this element.

### 4.2. Applications in Territorial Spatial Planning

WRSA and WRSU are used in territorial spatial planning and “Double Evaluation” to analyze the suitability for agricultural production and urban construction as a scientific basis for delineating agricultural and urban space. WRSA and WRSU should be used with other indicators. The case study of Baiyin City showed that although WRSA and WRSU can better identify suitable land for agricultural production and urban construction, the land resources suitable indicator is also irreplaceable. In addition, the indicators need to be used in cooperation with the water resources carrying capacity. Firstly, the overall quantity of the city and of farmland should be determined by the water resources carrying capacity. Then, the appropriate location should be determined through WRSA, WRSU, and other factors. In the territorial spatial planning of Baiyin City, WRSA and WRSU were used to divide the agricultural space and urban space, and to delimit permanent basic farmland and urban development boundaries. Agricultural land and urban land should be planned in areas with better water resources suitability, including river valley areas and water receiving areas of water diversion projects. In water resources unsuitable areas, the project of returning farmland to grassland should be promoted. Permanent basic farmland and urban development boundaries should not be designated in water resources unsuitable areas, and the intensity of cultivated land and constructed land should be reduced. These methods were carried out during the territorial spatial planning of Baiyin City and can be used as a reference for other regions.

### 4.3. Limitations

The results of water resource suitability evaluation are not completely consistent with the land use status. In total, 20.2% of the unsuitable area in Baiyin City are farmlands. According to the Third National Land Survey, these farmlands were mainly cultivated after 2010, resulting in an increase of 59.3% for cultivated land expansion in the past 10 years. The rapid increase in precipitation is the reason for the rapid growth of cultivated land, because the precipitation in recent years can satisfy the needs of non-irrigated farmland [71,72,73,74]. Since territorial spatial planning is long-term planning, the long-term annual average precipitation instead of the precipitation in recent years was used to delineate the unsuitable area. This is reasonable because most of the newly cultivated land in unsuitable areas were low yield farmland that were not suitable for permanent basic farmland. Of course, it does not mean that cultivated land and constructed land should not exist in water resources unsuitable areas. The indicator can be applied to territorial spatial planning to carry out agricultural production and urban construction layout in areas with high suitability for water resources, and to reduce the intensity of human activities in areas with low suitability. In the application, water source and parameters should be determined based on the specific conditions of the study area. For example, reservoirs, ponds, and groundwater are used as water sources in some areas, and economically developed metropolises can bear higher water supply costs, etc. It is necessary to conduct field investigations before the laboratory analysis to determine the parameters.

## 5. Conclusions

The layout of agricultural production and urban construction is an important part of China’s ongoing territorial spatial planning. Water resources are necessary for agricultural and urban development. Identifying spatial differences in the suitability of water resources can provide a scientific basis for delineating agricultural space and urban space and achieving an optimal layout of national land space. In this study, grid-scale indicators named WRSA and WRSU were constructed to evaluate the suitability of water resources for agricultural production and urban construction. Taking Baiyin City as a case, WRSA and WRSU were measured with an accuracy of a 20 m grid. According to the spatial analysis, the currently cultivated land and constructed land were mainly concentrated in suitable areas and relatively suitable areas, especially the irrigated farmland and the central city. The results of the Geodetector study also show that the WRSA and WRSU indicators have better explanatory power for cultivated land and construction land compared with traditional indicators such as water distance. Moreover, the coupled application with land resource suitability indicators can clearly explain the land-use spatial distribution. Therefore, it is reflected that the WRSA and WRSU can well characterize the ability of regional water resources to support agricultural production and urban construction. The results of this study have been applied to the territorial spatial planning of Baiyin City, which effectively promotes the delineation of urban space and agricultural space, as well as the delineation of urban development boundary and permanent basic farmland.

The grid-scale WRSA and WRSU could effectively describe the spatial differences of water resources suitability, which is difficult to distinguish using traditional regional-scale indicators. The indicators reflect the relative affordability of water supply costs and guides the spatial layout of both agricultural production and urban construction. The indicators can be easily applied to other regions, especially in arid and semi-arid regions, and also in hilly areas, where water supply suitability plays a restrictive role in agricultural production and urban construction. WRSA and WRSU are effective means of “determining regional functions by water resources “ in territorial spatial planning.

## Figures and Tables

**Table 1:** WRSA discriminant matrix based on CIA and precipitation.

		Yellow River	Other Rivers and Canals
CIA	0–0.5	suitable	relatively suitable
	0.5–1	relatively suitable	less suitable
	>1	less suitable (precipitation ≥ 400 mm)	
		unsuitable (precipitation < 400 mm)	

**Table 2:** WRSU discriminant matrix based on CIU.

	CIU		
	0~0.5	0.5~1	>1
Yellow River and central city trunk canal	suitable	relatively suitable	unsuitable
Other rivers and canals	relatively suitable	less suitable	unsuitable

**Table 3:** The distribution of cultivated land under different WRSA grades.

WRSA	Area (km^2^)	Cultivated Land Area (km^2^)	Proportion of Cultivated Land (%)	Irrigated Farmland Area (km^2^)	Proportion of Irrigation (%)
Suitable	381.0	133.7	35.1	131.6	98.4
Relatively suitable	3354.7	1329.2	39.6	944.0	71.0
Less suitable	3663.9	1718.0	46.9	83.8	4.9
Unsuitable	12,700.7	2570.4	20.2	135.6	5.3
Total	20,100.4	5751.2	28.6	1294.9	22.5

**Table 4:** The distribution of constructed land under different WRSU grades.

WRSU	Area (km^2^)	Constructed Land Area (km^2^)	Proportion of Constructed Land (%)
Suitable	1657.7	55.3	3.3
Relatively suitable	4184.5	68.7	1.6
Less suitable	1177.7	4.1	0.3
Unsuitable	13,075.7	13.7	0.1
Total	20,095.7	141.7	0.7

**Table 5:** Irrigated farmland density under different water and land resources suitability grades (%).

		Suitability of Land Resources for Agricultural Production					
		Suitable	Relatively Suitable	Moderately Suitable	Less Suitable	Unsuitable	Total
WRSA	Suitable	55.50	24.89	6.85	1.53	0.09	34.53
	Relatively suitable	41.86	19.70	4.94	0.97	0.46	28.14
	Less suitable	16.13	4.34	0.31	0.04	0.02	2.29
	Unsuitable	6.55	2.29	0.26	0.06	0.01	1.07
	Total	32.32	6.75	0.58	0.08	0.02	6.44

**Table 6:** Constructed land density under different water and land resources suitability grades (%).

		Suitability of Land Resources for Urban Construction				
		Suitable	Relatively Suitable	Less Suitable	Unsuitable	Total
WRSU	Suitable	7.06	2.10	0.35	0.05	3.34
	Relatively suitable	2.70	0.88	0.27	0.09	1.64
	Less suitable	0.90	0.27	0.08	0.06	0.35
	Unsuitable	0.44	0.14	0.04	0.01	0.10
	Total	2.39	0.51	0.08	0.01	0.71

**Table 7:** Factor interaction detection results of constructed land distribution.

	CIU	WRSU	Precipitation	Distance	Slope	Elevation
CIU	0.084					
WRSU	0.106	0.099				
Precipitation	0.289	0.322	0.079			
Distance	0.235	0.285	0.204	0.030		
Slope	0.366	0.409	0.243	0.192	0.110	
Elevation	0.249	0.313	0.312	0.254	0.174	0.097

## Data Availability

The data presented in this study are available on request from the corresponding author.
